# [NiEn_3_](MoO_4_)_0.5_(WO_4_)_0.5_ Co-Crystals as Single-Source Precursors for Ternary Refractory Ni–Mo–W Alloys

**DOI:** 10.3390/nano11123272

**Published:** 2021-12-01

**Authors:** Polina S. Serebrennikova, Vladislav Y. Komarov, Aleksandr S. Sukhikh, Svetlana P. Khranenko, Andrey V. Zadesenets, Sergey A. Gromilov, Kirill V. Yusenko

**Affiliations:** 1 Nikolaev Institute of Inorganic Chemistry, Lavrentiev Avenue 3, 630090 Novosibirsk, Russia; polina.buneeva2015@yandex.ru (P.S.S.); komarov_v_y@ngs.ru (V.Y.K.); a_sukhikh@niic.nsc.ru (A.S.S.); khranenko@niic.nsc.ru (S.P.K.); zadesenets@niic.nsc.ru (A.V.Z.); 2 Department of Physics, Novosibirsk State University, Pirogova Str. 2, 630090 Novosibirsk, Russia; 3 BAM Federal Institute for Materials Research and Testing, Richard-Willstätter Str. 11, 12489 Berlin, Germany

**Keywords:** complex salts, single-source precursors, molybdate anion, tungstate anion, X-ray diffraction, thermal decomposition, multicomponent nanostructured alloys

## Abstract

The co-crystallisation of [NiEn_3_](NO_3_)_2_ (En = ethylenediamine) with Na_2_MoO_4_ and Na_2_WO_4_ from a water solution results in the formation of [NiEn_3_](MoO_4_)_0.5_(WO_4_)_0.5_ co-crystals. According to the X-ray diffraction analysis of eight single crystals, the parameters of the hexagonal unit cell (space group *P*–31*c*, *Z* = 2) vary in the following intervals: a = 9.2332(3)–9.2566(6); c = 9.9512(12)–9.9753(7) Å with the Mo/W ratio changing from 0.513(3)/0.487(3) to 0.078(4)/0.895(9). The thermal decomposition of [NiEn_3_](MoO_4_)_0.5_(WO_4_)_0.5_ individual crystals obtained by co-crystallisation was performed in He and H_2_ atmospheres. The ex situ X-ray study of thermal decomposition products shows the formation of nanocrystalline refractory alloys and carbide composites containing ternary Ni–Mo–W phases. The formation of carbon–nitride phases at certain stages of heating up to 1000 °C were shown.

## 1. Introduction

The low-temperature preparation of binary alloys from single-source precursors has been studied in a large number of works in the last decade [1,2,3,4,5,6,7,8,9,10,11,12,13,14,15,16,17,18]. Binary alloys can be prepared for a large variety of refractory metals including Pt, Pd, Ir, Rh, and Os. The stoichiometry of the resulting alloys obtained by thermal decomposition is fixed by the composition of their precursors. The main drawback of this approach is the discrete composition of a single-source precursor which does not allow one to vary the alloy’s composition in a broad range. In the last decade, we developed a strategy to access not only binary phases but also ternary and multicomponent alloys with variable compositions using the co-crystallisation of isoformular isostructural coordination compounds [17]. As an example, [Ir(NH_3_)_5_Cl][OsCl_6_] was proposed as a precursor for *hcp*–Ir_0.5_Os_0.5_; [Ru(NH_3_)_5_Cl][IrCl_6_]—for Ir_0.5_Ru_0.5_; [Ru(NH_3_)_5_Cl]_2_[OsCl_6_]Cl_2_—for *hcp*–Os_0.33_Ru_0.67_. It has also been shown that isoformular compounds are isostructural and can be co-crystallized from water solutions. Such an important finding allowed us to first prepare binary *fcc*- and *hcp*-structured Ir–Ru alloys in the whole range of compositions according to the following generalised chemical equilibria:

*x*[Ir(NH_3_)_5_Cl]^2+^ + (1 − *x*)[Ru(NH_3_)_5_Cl]^2+^ + [IrCl_6_]^2−^ → [Ir*_x_*Ru_1−*x*_(NH_3_)_5_Cl][IrCl_6_] [Ir*_x_*Ru_1−*x*_(NH_3_)_5_Cl][IrCl_6_] → Ir_0.5+*x*/2_Ru_0.5−*x*/2_.

Subsequently, such an approach was extended to the ternary Ir–Ru–Os and multicomponent systems [19,20]:

*x*[Ir(NH_3_)_5_Cl]^2+^ + (1 − *x*)[RuNH_3_)_5_Cl]^2+^ + *y*[OsCl_6_]^2−^ + (1 − *y*)[IrCl_6_]^2−^ → [Ir*_x_*Ru_1−*x*_(NH_3_)_5_Cl][Os*_y_*Ir_(1−*y*)_Cl_6_];

thermal decomposition in H_2_ flow: [Ir*_x_*Ru_1−*x*_(NH_3_)_5_Cl][Os*_y_*Ir_(1−*y*)_Cl_6_] → Ir_0.5+*x*/2−*y*/2_Ru_0.5−*x*/2_Os*_y_*_/2_.

A single-source precursors’ approach seems to be extremely productive for the preparation of nano-porous refractory alloys. A possibility to vary composition using co-crystallisation makes the approach even more powerful to access alloys in a broad range of concentrations. The application of alloys with carbide-forming elements might be the next step for the design of multicomponent refractory ultrahard materials [21]. Nevertheless, systems with refractory metals such as Mo, W, and Ta have barely been investigated due to their high affinity to oxygen and the difficulties they present in being reduced from *M*O_4_^2−^ anions that are stable in water solutions. In the current study, we report an investigation of [NiEn_3_](MoO_4_)_0.5_(WO_4_)_0.5_ (En = ethylenediamine, H_2_NCH_2_CH_2_NH_2_) which can be obtained by the co-crystallization of two individual isoformular complex salts [NiEn_3_]WO_4_ and [NiEn_3_]MoO_4_. The crystal structures of both parent compounds were previously reported in [22,23]. Both salts are isostructural with a number of phases with the general formula [*M′*En_3_]*M″*O_4_ (*M′* = Co, Ni, Cu, Zn, Cd; *M″* = Cr, Mo, W, S). These phases can be considered as promising single-source precursors to access binary alloys as well as composite carbide and oxide materials using their thermal decomposition in a reductive (hydrogen) and oxidative (oxygen and air) atmosphere. Thermal decomposition can be performed not only by using a solid/gas interface but also solid-state reaction. Thus, [NiEn_3_]MoO_4_ can be decomposed using LiH as a reduction agent in solid state (in a He flow as protective atmosphere) at 1000 °C. Decomposition results in a formation of MoNi_4_, Mo_2_C, and Ni_2_Mo_4_C*_x_* mixture. The thermal decomposition of [NiEn_3_]WO_4_ in H_2_ flow as a reducing agent at 410 °C results in a formation of an *hcp*-structured Ni(C*_x_*) interstitial alloy mixed with *bcc*-W_1−*x*_Ni*_x_* [24]. At 510 °C, an additional *fcc*-Ni_1−*x*_W*_x_* binary alloy was obtained. Double-carbide Ni_2_W_4_C was detected above 660 °C as was Ni_6_W_6_C at 860 °C. These phases were proposed as an active catalyst for the synthesis of carbon nanotubes and carbon nanofibres [25,26] and as cathode materials for the synthesis of hydrogen by water electrolysis [27]. It is expected that the thermolysis of the co-crystallization product (CCP) of [NiEn_3_]MoO_4_ and [NiEn_3_]WO_4_ can lead to the formation of ternary alloys and carbides.

In the current study, a series of [NiEn_3_](MoO_4_)_1−*x*_(WO_4_)*_x_* as single-source precursors for ternary refractory alloys were synthetized from water solutions. The slow co-crystallisation of isoformular isostructural [NiEn_3_]MoO_4_ and [NiEn_3_]WO_4_ from water solution results in the formation of not a single-phase precipitate but a number of single crystals with large variations in their composition. The quality of the resulting single crystals may lead us to perform a single-crystal X-ray diffraction study to refine the chemical composition of each individual crystal. Individual crystals were used as single-source precursors for ternary Ni–Mo–W alloys after their decomposition above 1000 °C.

## 2. Materials and Methods

Synthesis of [NiEn_3_](MoO_4_)_0.5_(WO_4_)_0.5_: Here, 1 mmol Ni(NO_3_)_2_·6H_2_O was dissolved in 15 mL H_2_O. Then, 1M NaOH water solution was added to form colloidal Ni(OH)_2_ precipitate. Formed solid Ni(OH)_2_ was centrifugated (3 min, 3000 rpm) and washed 3 times with a 1:1 water:ethanol mixture. Fresh Ni(OH)_2_ precipitate was dissolved in ethylenediamine, formed a dark red precipitate after 15–20 min, and was mixed with Na_2_WO_4_·H_2_O (0.5 mmol, 0.165 g) and Na_2_MoO_4_ (0.5 mmol, 0.165 g) in 5 mL water. After intensive stirring, the reaction mixture was placed in a desiccator with solid NaOH as a drying agent. After 2 days at room temperature, violet crystals were collected. The yield was 85–90%.

An in-house X-ray powder diffraction study was performed at room temperature using a SHIMADZU powder XRD-7000 diffractometer (Shimadzu, Kyoto, Japan) (CuKα—radiation, λ_Kα1_ = 1.5406 Å, Ni-filter) in the range from 5° to 70° 2θ with a step of 0.03°. The salts-precursors and [NiEn_3_](MoO_4_)_0.5_(WO_4_)_0.5_ crystals were slightly ground in an agate mortar with heptane and LaB_6_ (SRM660a, *a* = 4.1569 Å) as the internal standard. The samples were placed as a thin layer on the polished side of a standard quartz cuvette. The diffraction pattern was indexed using single crystal data obtained for parent [Ni*En*_3_]MoO_4_ (sp. gr. *P*3-1*c*, cell parameters: *a* = 9.2425(5) Å, *c* = 9.9601(5) Å). The positions of single reflexes were fitted as Lorentz functions using the Fityk software (Marcin Wojdyr, Institue of High Pressure Physics, Warsaw, Poland) [28]. A piecewise linear polyline dependence Δ2θ(2θ) was constructed from the positions of K_a1_-components of LaB_6_ reflexes, and then used to apply corrections to the measured 2θ position of 11 reflexes corresponding to the studied salts. The unit cell parameters (UCPs) obtained by the least squares refinement were *a* = 9.2451(5), *c* = 9.9682(8) Å (FWHM~0.25) for [NiEn_3_](MoO_4_)_0.5_(WO_4_)_0.5_, *a* = 9.2348(4); *c* = 9.9199(4) Å (FWHM~0.1) for [NiEn_3_]MoO_4;_ and *a* = 9.2601(3), *c* = 9.9781(5) Å (FWHM~0.2° 2θ) for [NiEn_3_]WO_4_ (sp. gr. *P*3-1*c*). All FWHM values are given for reflexes (302), 2θ~38°.

**Figure 1:**
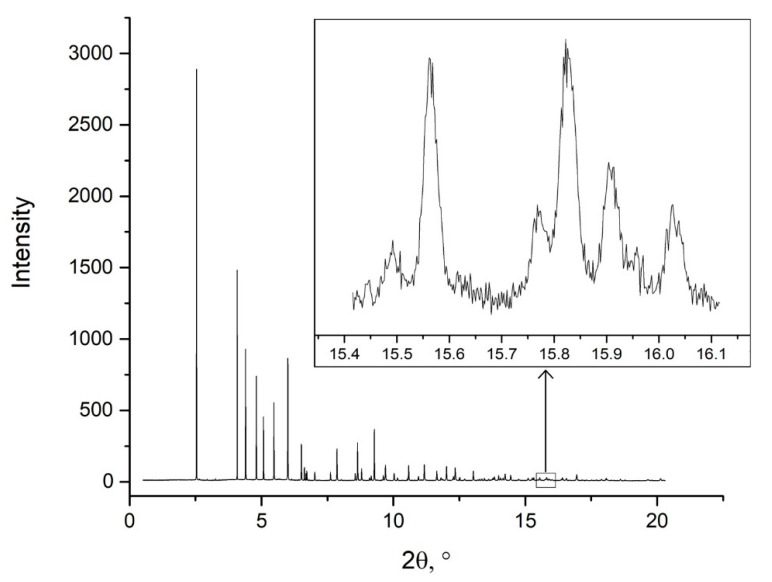
High-resolution synchrotron X-ray powder diffraction data (λ = 0.354395 Å) of [NiEn_3_](MoO_4_)_0.5_(WO_4_)_0.5_. The inserted diagram shows the splitting of lines at the far angles’ of diffraction.

High-resolution synchrotron X-ray powder diffraction (PXRD). Diffraction patterns for powdered [NiEn_3_](MoO_4_)_0.5_(WO_4_)_0.5_ and salts-precursors were recorded at a high-resolution ID22 beamline at the ESRF (λ = 0.354395 Å) [29]. The station was equipped with a nine-channel detector with silicon crystal analysers (each channel is offset relative to the next one by 2° 2θ, data from each channel were independently recorded, and the information was then summed with an integration step of 0.002°). The beam size was 2 × 1 mm^2^. During the shooting process, the detector was moved in continuous scanning mode around the sample axis. The sample in powder form was placed and sealed in 0.2 mm borosilicate glass mark tubes (Hilgenberg GmbH, Malsfeld, Germany). During the measurement, samples were axially rotated to provide an additional misdirection of the crystallites. The FWHM of single reflexes was 0.04° 2θ at 10–15° 2θ. A slight splitting of the diffraction lines at the high diffraction angles was recorded. The splitting indicates the heterogeneity (see inserted diagram in Figure 1) of the crystallite composition. Similar broadness was previously detected using in-house PXRD, but the synchrotron study gives a much higher number of individual reflexes to be fitted (up to 53) with a much higher instrumental resolution. For the refinement of cell parameters, diffraction profiles were fitted using the Gauss function (Figure 1). Average cell parameters were refined as a = 9.2409(2), c = 9.9576(3) Å (FWHM~0.012) for [NiEn_3_](MoO_4_)_0.5_(WO_4_)_0.5_, *a* = 9.2440(5), *c* = 9.9607(6) Å (FWHM~0.015) for [NiEn_3_]MoO_4_ and *a* = 9.2707(2), *c* = 9.9863(4) Å (FWHM~0.012° 2θ) for [NiEn_3_]WO_4_. All FWHM values are given for (112) reflexes at 2θ~6°.

The single-crystal X-ray diffractions (SCXRD) of individual single crystals (~0.1–0.2 mm) were performed using a Bruker Duo and Bruker X8 (MoKα—radiation, λ_Kα1_ = 0.70932 Å, graphite monochromator, focusing collimator 0.60FC, four—axial goniometer, CCD detector, resolution 512 × 512, pixel size—120 microns). The temperature of each investigated single crystal was kept at 298(2) K during SC-XRD analysis using an Oxford Cryosystems Cobra (Bruker DUO, Bruker AXS GmbH, Karlsruhe, Germany) and Oxford Cryosystems Cryostream 800plus (Bruker X8, Bruker AXS GmbH, Karlsruhe, Germany) open-flow nitrogen coolers. The integration of experimental intensities accounting for absorption was performed using the APEX2 software package [30]; the structures were solved using the ShelxT [31] and refined using the ShelxL software package [32] as well as the Olex2 graphical interface [33]. For this study, 7 single crystals were selected. The cell parameters of the studied single crystals are given in Table 1. For all crystals, the occupancies of the metal sites in anions were refined.

Structural data for [NiEn3](MoO_4_)_1−x_(WO_4_)_x_ single crystals with different values of *x* (№1 with *x* = 0.487(3), №5 with *x* = 0.673(4), №6 with *x* = 0.824(4) and №7 with *x* = 0.924(5)) were deposited into the CCDC database under 2113885, 2113886, 2113887, and 2113888, respectively. All data are freely available online at https://www.ccdc.cam.ac.uk/ (accessed on 1 December 2021).

The thermal decomposition of [NiEn_3_](MoO_4_)_0.5_(WO_4_)_0.5_ was performed according to the protocol described in [24]. Briefly, several individual single crystals were mixed with LiH powder (1:1 volume ratio) and placed in a molybdenum crucible (outer diameter 3 mm; inner diameter 2 mm). A helium flow (99.995% purity) was used as protective atmosphere. Heating up to 1100 °C was carried out with 600°/min ramp, and the sample was kept at the final temperature for 1 min. The admixture of Li_2_O was washed with distilled water. A phase analysis was performed using a search-match in Powder Diffraction Files and International Crystal Structure Database [34,35].

The thermal decomposition of individual crystals was also performed in hydrogen flow. For the thermal decomposition, individual needle-shaped 1–2 mm single crystals were selected. Crystals №4 and №5 were heated in a hydrogen flow of up to 1000 °C with a heating rate of 10°/min and kept at the final temperature for 10 min. Crystals №2 and №3 were heated up to 1000 °C with a 10°/min ramp and tempered for 8 h at constant temperature. Mo/W ratios were obtained after the refinement of the single crystal X-ray diffraction data collected for 0.2 mm fragments of the same crystals.

The X-ray study of thermolysis products was carried out in the transmission geometry using a Bruker D8 Venture diffractometer (microfocus tube Incoteac IμS 3.0, Cu*K*α radiation, PHOTON 3 detector, resolution 768 × 1024, pixel size—135 microns; *D* = 69 mm; 2θ_D_ = 45°). The detector position errors—distance D, angle 2θ and slopes were obtained from Si powder (SRM-640, *a* = 5.4309 Å) measurements and refined using the Dioptas software [36]. Cell parameters were refined from an additional measurement with *D* = 69 mm, 2θ_D_ = 120°. The obtained phase composition and cell parameters are given in Table 2.

## 3. Results and Discussion

**Figure 2:**
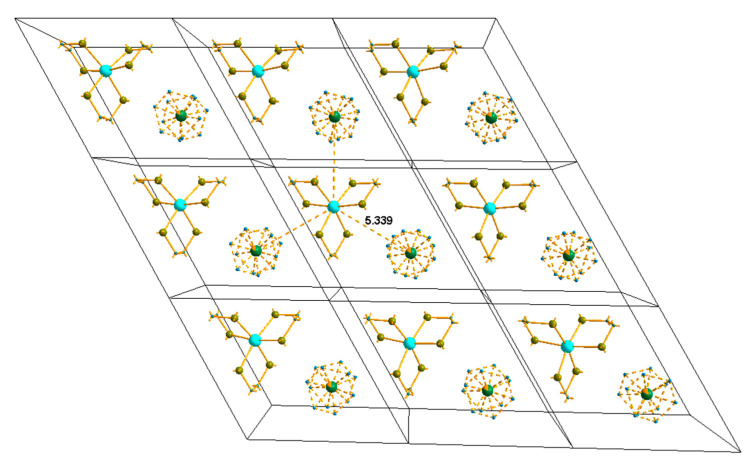
Model of anion disordering in a layer perpendicular to the *c* axis for crystal No 6. The Mo/W ratio is shown as a pie chart in the place of the corresponding atom (green is W and yellow is Mo).

[NiEn_3_]MoO_4_ and [NiEn_3_]WO_4_ are isoformular and isostructural with close hexagonal cell parameters. Their crystal structures contain isolated octahedral cations [NiEn_3_]^2+^ and tetrahedral *M*O_4_^2−^ anions (Figure 2). In the plane of the figure, each cation is surrounded by three anions with three identical *M*…Ni distances of 5.34 Å. Additionally, in the coordination surroundings of the cation—the trigonal bipyramid—two additional anions are involved in the direction of the *c* axis at the distances of 4.979 Å. Anions have similar surroundings. Thus, in the initial phase—the precursor—the metal atoms are mixed at the nanoscale.

All previous studies of multimetallic complex salts have been performed using powder X-ray diffraction. All multimetallic salts were prepared from isoformular isostructural salts with very similar cell parameters. In all studies, single-phase nature with the random metallic distribution of co-crystallised precursors similar to substitution in metallic solid solutions was postulated without an extensive analysis of the precursor’s crystal structures and their possible inhomogeneity. Our preliminary PXRD study of bulk [NiEn_3_](MoO_4_)_0.5_(WO_4_)_0.5_ material shows relatively broad diffraction lines. Such line-broadness might be explained by the low crystallinity of starting materials similar to those of previous works with multimetallic compounds and relatively low solubility [19,20,37]. In the current study, powder was prepared from ideal relatively large single crystals (up to several mm), where sample-specific line broadness seems to be negligible. It should also be noted that the FWHM of the lines in the diffraction pattern of the CCP turned out to be noticeably larger than the FWHM of the precursor salts. Theoretically, this broadening can be associated with the overlap of adjacent lines during the formation of a mechanical mixture of the initial phases which cannot be resolved due to the insufficient quality of laboratory diffraction data. This means that, according to the data of the laboratory powder diffraction study, it is impossible to conclude that the CCP is homogeneous. The powder diffraction study at the ID22 ESRF beamline allowed us to perform PXRD data collection using an instrument with one of the narrowest instrumental resolutions [26]. The average FWHM of the lines in the three obtained diffraction patterns coincide well and are smaller than in the case of the laboratory powder diffraction study. A visible splitting of the diffraction lines at the high-angle region was recorded. The splitting indicates the possible multiphase content of the powder. High-angle diffraction lines allow us to refine average cell parameters using the Gauss profile function as average *a* = 9.2409(2), *c* = 9.9576(3) Å (splitting was not taken into account while estimating the UCPs).

To prove our findings, several single crystals collected from the same synthesis were investigated. Each individual ideal single crystal seems to be homogeneous without a visible line broadness specific for low-crystalline materials. Nevertheless, there is significant variation in cell parameters between individual single crystals collected from the same bunch: *a* = 9.2332(3)−9.2566(6), *c* = 9.9512(12)−9.9753(7) Å; Mo/W ratio obtained from the refinements of site occupancies changes from 0.513(3)/0.487(3) to 0.078(4)/0.895(9) (see details in Table 1).

The co-crystallisation from homogeneous equimolar solution occurs with large variations in unit cell parameters and crystal composition. Based on our findings, we can assume that the co-crystallisation of mixed salts with relatively low solubility such as [Ru(NH_3_)_5_Cl][IrCl_6_] and [Ru(NH_3_)_5_Cl][IrCl_6_], where solubility can be estimated as 10^−3^–10^−4^ mol/L at room temperature in pure water, results in the fast crystallisation of homogeneous powder even from diluted solutions. In such a case, the concentration of the water solution directly controls the composition of the solid phase without the large enrichment of one component. The solubility of [NiEn_3_]MoO_4_ and [NiEn_3_]WO_4_ seems to be much higher and their crystallisation occurs from solutions with a relatively high concentration and viscosity. As a result, nucleation might occur with spontaneous enrichment with one component. A large number of seed crystals with variations in their composition form a large set of crystals with broad variations in their cell parameters and properties. Such a situation seems to be far from equilibrium, but further equilibration needs fast exchange between crystals through solution which probably need a long time with intensive stirring and/or heating. The described phenomena should play a critical role in the application of multimetallic salts as single-source precursors for multimetallic alloys. Indeed, if each crystal has its own composition and own crystal structure, inhomogeneous single-source precursors should form inhomogeneous alloys after decomposition.

**Figure 3:**
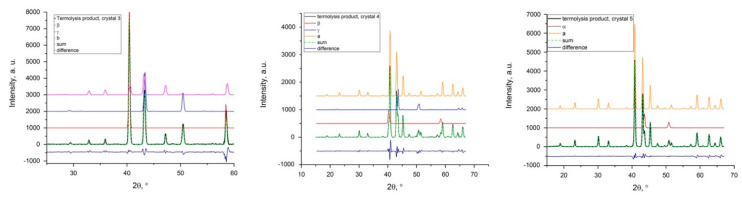
The results of the profile matching and cell parameters’ refinement diffraction pattern corresponding to the thermolysis of crystals №3 (**left**), №4 (**middle**), and №5 (**right**) in H_2_ (in-house single crystal diffractometer, MoKα−radiation, λ_Kα1_ = 0.70932 Å). Metallic products contain *fcc*−structured alloy (α), *bcc*−structured alloy (β), (Mo,W)Ni_4_ (γ), Ni_2_(Mo,W)_3_N*_x_* (a), and Ni_6_(Mo,W)_6_C_x_ (b) phases. Inserts show original 2D diffraction images used for the integration of the patterns.

Several individual single crystals were decomposed at 1000 °C in a H_2_ flow (Table 2). The main phases in samples №4 and №5 were a double nitride based on the cubic Ni_2_Mo_3_N [35; № 50815] or isostructural Ni_2_W_3_N [35; № 86170]. The measured values of cubic cell parameter *a* and atomic volume (*V*_m_) are closer to pure Ni_2_Mo_3_N. Nevertheless, one can also assume the formation of a non-stoichiometric phase with a lower nitrogen content. These ambiguities in the composition of double phases do not allow us to perform more precise quantitative phase analysis based on PXRD data. In addition, samples №4 and №5 revealed admixtures of *fcc*-structured alloys with cell parameters which were significantly larger in comparison with pure Ni, which is an indication of the formation of Ni alloyed with Mo and W (Figure 3).

Additionally, sample №4 contains several minor admixtures such as a *bcc*-structured Mo- (or W-)-based alloy as well as (Mo,W)Ni_4_ intermetallics. The *bcc*-phase has a cell parameter between Mo and W. According to the Ni–Mo and Ni–W binary phase diagrams, the maximal Ni solubility in the *bcc*-structured phase is 2 at. %. The Mo/W = 67/33 ratio in the *bcc*-phase can be estimated using Zen’s law.

The atomic volume is defined as a ratio between the unit cell volume, V, and the number of atoms per unit cell, *Z* (*Z* = 2 for *bcc* and *Z* = 4 for *fcc-*structured alloys). According to Zen’s law [38], the atomic volumes, *V*_M_ = *V*/*Z* (Å^3^·atom^−1^), for *bcc*–*fcc* bimetallic alloys, should follow a nearly linear dependence on composition with a relatively small positive or negative deviation [39]. For the (Mo,W)Ni_4_ intermetallic phase, the Mo/W ratio was also estimated according to Zen’s law based on atomic volumes (*V*_M_) of MoNi_4_ and WNi_4_. The formation of partially disordered intermetallics and/or the inclusion of C and N atoms should be considered. The MoNi_4_ phase was observed as a product of the thermal decomposition of [NiEn_3_]MoO_4_ with LiH at 1000 °C. The cell parameter *a* = 5.691 Å was significantly larger but the *c* = 3.577 Å and *V*_M_ = 11.58 Å^3^·atom^−1^ were significantly smaller in comparison with the reference values refined as *a* = 5.683, *c* = 3.592 Å, and *V*_M_ = 11.66 Å^3^·atom^−1^.

The simultaneous decomposition of 10–15 [NiEn_3_](MoO_4_)_0.5_(WO_4_)_0.5_ crystals using LiH (~1:1 volume ratio) as a reducing agent at 1100 °C led to the formation of a three-phase mixture. The *Fcc*-structured phase based on the Ni lattice, *bcc*-structured phase based on Mo(W), as well as the ternary or four-component Ni_2_Mo_4_(W)C*_x_* carbide were observed (Table 2). In [24], a mixture of *fcc*-alloy (*a* = 3.583 Å) and Ni_2_W_4_C*_x_* (*a* = 11.200 Å) was observed after the thermal decomposition of [NiEn_3_]WO_4_ mixed with LiH under similar conditions. For [NiEn_3_]MoO_4_ mixed with LiH (He flow, 1050 °C, 1 min), a mixture of Mo_2_C, MoNi_4_ and Ni_2_Mo_4_C*_x_* phases was obtained.

After 8 h at 1000 °C, the phase composition of samples №2 and №3 significantly changed. The *bcc*-phase based on Mo (or W) and (Mo,W)Ni_4_ were found to be the main phases. The intermetallic phase has abnormally large (relative to MoNi_4_ and WNi_4_) *V*_M_ values, which might be explained by the partial replacement of Ni by Mo and W, as well as the inclusion of C and N into the lattice. The Ni_6_(Mo,W)_6_C phase was also detected as minor admixture in samples №2 and №3.

The unstable phase composition of samples obtained by holding the CCP at 1000 °C for 10 min can be explained by a small quantity of samples. This entails the irregularity in heat distribution inside the oven and as a result, the faster heating of sample №4 and the earlier appearance of β- and γ-phases. As shown in Table 2, the phase composition and the UCP satisfactorily coincide while increasing the exposure time to 480 min at 1000 °C. Subsequently, only these results are considered.

**Figure 4:**
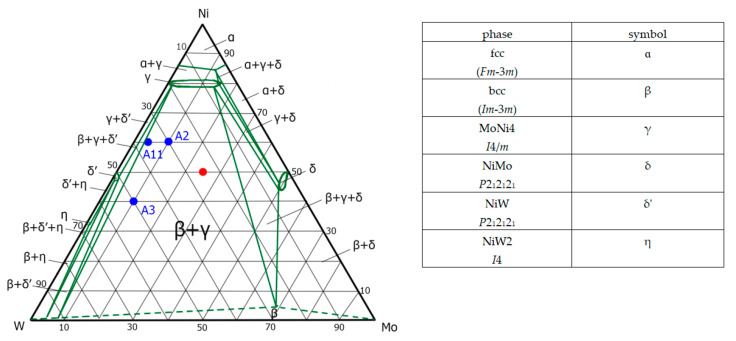
Ternary W–Mo–Ni (1000 °C isothermal cross-section). The red circle highlights the area of the initial atoms’ ratio Ni/Mo/W studied in this work; the blue circles highlight the areas of the initial metal ratio of the samples from [44].

Figure 4 shows the cross-section of the ternary phase diagram for a temperature of 1000 °C, constructed of ternary diagrams of the Ni–Mo–W system [40,41,42,43,44] and binary diagrams [45,46], as well as taking into account the available experimental data [47,48]. The most recent Mo–Ni–W ternary phase diagram at 1000 °C is given in [44]. It was constructed by a combination of calculations using the CALPHAD and a limited array of experimental data (UCP and metal ratios) [43]. Figure 4 shows the regions of the existence of individual phases and their mixtures, as well as the regions of the compositions of precursors corresponding to our work and that of [44]. On the ternary phase diagram [40], the region corresponding to the specified compositions of precursors assumes the formation of a mixture of only α-and β-phases, while the (Mo,W)Ni_4_ (γ-phase) is present on the binary Ni–Mo phase diagram only up to 870 °C. However, its presence in thermolysis products when they are heated up to 900 °C is shown in [44], and its appearance when heated up to 1000 °C is demonstrated in our work, so the corresponding area was plotted in Figure 4. Additionally, in the diagram for 1000 °C in [44], there is a region α + β + γ, which does not correspond to the data obtained in the current study. However, due to its rapid migration following an increase in the proportion of Mo with a decrease in temperature from 1000 to 900 °C and the presence of an error in setting the temperature in our experiment, this area cannot be taken into account while constructing the diagram.

Table 3 shows a comparison of the data obtained by us with the data presented in [44]. While comparing the results of [44] with our data (Table 2), the presence of UCP differs in the second–third decimal place for *bcc*—and (Mo,W)Ni_4_ phases can be seen. The values of point A11 are closest to our UCP, however, the initial ratios of the samples from [44] significantly differ from ours.

One should also take into account a possible formation of ternary Mo–Ni–C [49], W–Ni–C [50] carbides, and quaternary Mo–Ni–W–C [51].

## 4. Conclusions

Access to refractory alloys and ceramics based on W and Mo requires high temperature annealing or melting above 3000 °C. The works [52,53] show the formation of such alloys as a result of mechanochemical treatment for 20 h. Single-source precursors’ strategy allows us to prepare not only ternary Mo–W–Ni alloys but also carbides and nitrides at relatively low temperatures below 1000 °C. Powder and single-crystal XRD study suggests that each homogeneous single crystal shows a large variety in composition, and as a result, various cell parameters. In this study, we show that it is important to more carefully study the products of co-crystallization, which in the future, are supposed to be applied as precursors in the production of metal phases.

The thermal decomposition of [NiEn_3_](MoO_4_)_0.5_(WO_4_)_0.5_ in hydrogen flow at 1000 °C results in a formation of ternary Ni–Mo–W *fcc*- and *bcc*-structured alloys mixed with four component carbides and nitrides. Decomposition using solid LiH at 1000 °C results in a formation of ternary alloys mixed with four-component cubic Ni_2_(Mo,W)_4_C*_x_* carbide, which does not form in H_2_ flow, and no ordered phases were detected.

## Figures and Tables

**Table 1:** Results of the study of [Ni*En*_3_](MoO_4_)_1−*x*_(WO_4_)*_x_* individual single crystals *.

Number of the Crystal	1	2	3	4	5	6	7
CCDC database number	2113885	–	–	–	2113886	2113887	2113888
*a*, Å	9.2332(9)	9.2363(5)	9.2344(5)	9.2430(5)	9.2566(6)	9.2475(6)	9.2425(1)
*c*, Å	9.9512(12)	9.9582(5)	9.9577(6)	9.9590(5)	9.9753(7)	9.9639(8)	9.9682(2)
*V*, Å^3^	734.7(2)	735.7(1)	735.6(1)	736.8(1)	740.2(1)	737.9(1)	737.4(2)
*D*_x_, g/cm^3^	1.996	2.007	2.014	2.030	2.056	2.122	2.163
θ interval, °	2.55–30.61	4.87–30.49	2.05–30.49	2.54–28.67	2.54–30.23	2.54–30.61	2.04–30.46
Completeness, %	100	95.8	98.67	97.35	100	100	100
*N*_parameters_	43	43	43	43	43	43	43
*S*-factor by *F*^2^	1.047	1.119	1.105	1.168	1.001	1.030	1.075
*R*_1_ [*I* > 2σ(*I*)]	0.0133	0.0153	0.0168	0.0154	0.015	0.0114	0.0146
*wR*_2_ [*I* > 2σ(*I*)]	0.0299	0.0308	0.0279	0.0347	0.0307	0.0264	0.0287
*R*_1_ (all data)	0.0157	0.0174	0.0210	0.0175	0.0177	0.0133	0.0175
*wR*_2_ (all data)	0.0310	0.0315	0.0286	0.0357	0.0314	0.0269	0.0295
*x*	0.487(3)	0.517(4)	0.536(4)	0.583(5)	0.673(4)	0.824(4)	0.924(5)

* [NiEn_3_]MoO_4_: *a* = 9.2425(5) Å, *c* = 9.9601(5) Å, *V* = 736.84(9) Å^3^, *Z* = 2, sp. gr. *P*-31*c*; [NiEn_3_]WO_4_: *a* = 9.2641(3) Å, *c* = 9.9817(3) Å, *V* = 741.89(4) Å^3^, *Z* = 2, sp. gr. *P*-31*c*.

**Table 2:** The results of the X-ray phase analysis of thermal decomposition products (the crystal numbers correspond to the numbers from Table 1).

Conditions Phase	T, °C t, min Gas	1000 1 He (LiH)	1000 480 H_2_	1000 480 H_2_	1000 10 H_2_	1000 10 H_2_
N of sample.		№1	№2	№3	№4	№5
Ni/Mo/W mol. %		50/26/24	50/24/26	50/23/27	50/21/29	50/23/27
fcc (α) (*Fm-*3*m*)	*a*, Å	3.592(2)	–	–	3.587(2)	3.593(2)
	*V_M_*, Å^3^	11.586			11.538	11.596
bcc (β) (*Im-*3*m*)	*a*, Å	3.148(2)	3.154(2)	3.154(2)	3.153(2)	–
	*V_M_*, Å^3^	15.598	15.688	15.688	15.672	
(Mo,W)Ni_4_ (γ) (*I*4/*m*)	*a*, Å	–	5.719(4)	5.719(4)	5.701(4)	–
	*c*, Å		3.588(2)	3.609(2)	3.574(2)	
	*V_M_*, Å^3^		11.735	11.804	11.616	
Ni_2_(Mo,W)_3_N*_x_* (a) (*P*4_1_32)	*a*, Å	–	–	–	6.633(4)	6.634(4)
	*V_M_*, Å^3^				14.592	14.598
Ni_6_(Mo,W)_6_C*_x_* (b) (*Fd*-3*m*)	*a*, Å	–	10.875(6)	10.884(6)	–	–
	*V_M_*, Å^3^		14.615	14.651		
Ni_2_(Mo,W)_4_C*_x_* (c) (*Fd*-3*m*)	*a*, Å	11.228(6)		–	–	–
	*V_M_*, Å^3^	16.085				

Ni: *a* = 3.5239 Å, *V_M_* = 10.940 Å^3^ [35; № 8688]; Mo: *a* = 3.1472 Å, *V_M_* = 15.586 Å^3^ [35; № 76147]; W: *a* = 3.1648 Å, *V_M_* = 15.849 Å^3^ [35; № 43421]; MoNi_4_: *a* = 5.683, *c* = 3.592 Å, *V_M_* = 11.61 [35; № 644017]; WNi_4_: *a* = 5.730, *c* = 3.553 Å, *V_M_* = 11.66 [34; № 03-065-2673]; Ni_2_Mo_3_N: *a* = 6.6340 Å, *V_M_* = 14.598 Å^3^ [35; № 50815]; Ni_2_W_3_N: *a* = 6.663 Å, *V_M_* = 14.790 Å^3^ [35; № 86170]; Ni_6_Mo_6_C: *a* = 10.891 Å, Z = 8, *V_M_* = 13.456 Å^3^ [35; 618328]; Ni_6_W_6_C: *a* = 10.873 Å, Z = 8, *V_M_* = 14.607 Å^3^ [35; 618588]; Ni_2_Mo_4_C: *a* = 11.250 Å, Z = 16, *V_M_* = 14.832 Å^3^ [35; 76137]; Ni_6_W_6_C: *a* = 11.226 Å, Z = 16, *V_M_* = 14.607 Å^3^ [35; 199847].

**Table 3:** The results of the X-ray phase analysis of thermal decomposition products.

Phase		A2 [43]	A3 [43]	A11 [43]	Current Work	
					№2	№3
Ni/Mo/W at. %		60/10/30	40/10/50	60/4/36	50/24/26	50/23/27
*bcc* (β) (I*m*-3*m*)	*a*, Å	3.14845	3.16408	3.16351	3.154(2)	3.154(2)
	*V_M_*, Å^3^	15.605	15.838	15.830	15.688	15.688
(Mo,W)Ni4 (γ) (*I*4/*m*)	*a*, Å	5.73774	5.70910	5.71754	5.719(4)	5.719(4)
	*c*, Å	3.55725	3.57707	3.57772	3.588(2)	3.609(2)
	*V_M_*, Å^3^	11.711	11.659	11.696	11.735	11.804
